# Influence of low-level laser therapy on implant stability in implants placed in healed sites: a randomized controlled trial

**DOI:** 10.1186/s40729-021-00331-0

**Published:** 2021-06-01

**Authors:** Mateus de Azevedo Kinalski, Bernardo Antonio Agostini, Cesar Dalmolin Bergoli, Mateus Bertolini Fernandes dos Santos

**Affiliations:** 1 Graduate Program in Dentistry, Federal University of Pelotas, Pelotas, Rio Grande Brazil; 2 Graduate Program in Dentistry, Meridional Faculty/IMED, Passo Fundo, Brazil

**Keywords:** Dental implants, Gallium aluminum arsenide lasers, Osseointegration, Randomized clinical trial

## Abstract

**Background:**

The present study aims to assess the influence of low-level laser therapy (LLLT) on stability in implants placed in healed sites.

**Material and methods:**

The present study followed the SPIRIT statement and is reported according to CONSORT. Patients were randomly allocated to LLLT or control groups. LLLT consisted in the application of 808-nm GaAlA laser applied before the preparation of the implant bed and after suturing (80 seconds; 11J/cm^2^). Implant stability quotient (ISQ) and the distance between the implant platform to the alveolar bone crest (millimeters) were assessed at implant placement (T_0_) and the abutment selection phase (4–6 months, T_a_).

**Results:**

A total of 64 implants were placed in 33 patients. The insertion torque ranged from 10 to 70 N.cm (mean 43.23; SD ±16.82). The T_0_ ISQ ranged from 18 to 95.5 (mean 61.7; SD ±18.23) and the crestal bone radiographic distance was 2.03 mm (SD±1.27). At T_a_, the ISQ ranged from 39 to 90 (mean 64.2; SD±9.84), and the mean crestal bone radiographic loss was 1.70mm (SD±1.65). However, no differences were observed when LLLT and control groups were compared with ISQ difference (T_a_–T_0_; *p*=0.598) or radiographical peri-implant alterations (*p*=0.531).

**Conclusion:**

LLLT did not influence the implant stability in implants placed in healed sites compared to a control group.

**Trial registration:**

ReBEC, RBR-35TNJ7. Registered May 23, 2018

## Background

Dental implants are the gold standard for replacing missing teeth. It can be prescribed from a single tooth to full-arch rehabilitation and can be placed at the same appointment of tooth extraction or after proper healing of an extraction socket [1]. In this perspective, the placement of dental implants in healed sites presents more favorable outcomes and greater survival rates compared to implants placed in fresh extraction sockets. However, no differences are observed when considering implant stability between these techniques [2]. Although the survival and success rates of it are notably high [3], there is still room for improvement in the survival and success rates, as well as, for improving implant stability at implant placement in order to achieve osseointegration without a significant reduction in the peri-implant marginal bone.

Among the studies investigating the improvement of implant stability, undersized site preparation, flapless surgery, and the application of low-level laser therapy (LLLT) have been suggested [4–7]. In regard to LLLT, infrared wavelength light is applied to the surrounding tissues, in order to reduce inflammatory responses, bio-stimulate osteoblastic activity around the application area, and consequently enhance bone formation [8–12]. A systematic review concluded that several animal studies have indicated that LLLT could facilitate hard and soft tissue regeneration, promoting osseointegration and improving implant stability [13]; however, there is still a gap in the literature regarding pieces of evidence obtained from clinical studies. Recently published randomized controlled trials on this topic [11, 14] have reported controversial results in regard to implant stability enhancement when LLLT was applied compared to a control group.

Considering that there is no sufficient evidence to support or refute that LLLT have a positive effect on implant stability in humans, the randomized controlled trial was designed to assess the influence of LLLT on implant stability in implants placed in healed tooth extraction sites. The null hypothesis tested was that there would be no difference in implant stability considering the intervention with LLLT in implants placed in healed bone sites compared to a control group.

## Materials and methods

### Study design

This is a prospective equivalence randomized controlled trial with parallel groups blinded to the evaluators designed according to the SPIRIT statement [15] and reported following CONSORT guidelines [16]. The study protocol was in accordance the Helsinki Declaration of 1975, as revised in 2000, and was approved by the institutional ethics committee (Protocol 2.369.402), and the trial was registered prior to beginning (ReBEC TRIAL: RBR-35TNJ7). The study was conducted from June 2017 to June 2019.

### Eligibility criteria

The adopted inclusion criteria were (1) At least 21 years old; (2) have at least one healed (≥ 6 months) tooth extraction site requiring rehabilitation; (3) adequate bone dimensions for implant placement without the need for guided bone regeneration procedures; (4) good general health, which allows for dental implant surgery; (5) availability for dental appointments at the institution; and (6) signed informed consent given by the patient.

Exclusion criteria are (1) any uncontrolled systemic diseases that prevent surgery for dental implant placement (e.g., hypertension, metabolic bone disease, diabetes); (2) need of tooth extraction in the region; (3) less than 6 months after tooth extraction; (4) need for guided bone regeneration or sinus lift for implant placement; and (5) history of radiation therapy in the head and neck.

### Randomization and allocation concealment

Patients were included in the study after fulfilling all the eligibility criteria and signing the informed consent form for participation and permission to use obtained data for research purposes. Patients were randomly allocated according to the type of treatment (control group: conventional protocol for implant placement and intervention group: LLLT). The allocation process considered the implants as units and was performed using the Random Allocator software® in blocks of 6. To ensure concealment of randomization, consecutively numbered brown envelopes were used, with the following intervention draws: CONTROL and LLLT and only one researcher was involved in this process. The team of surgeons only became aware of the intervention at the time of surgery, when the envelope was opened by a blinded researcher.

### Clinical procedures

All surgeries were carried out by the same group of surgeons who were specialists in oral implantology and performed all surgeries with the reflection of a flap and direct access to the bone tissue. Grade 4 titanium implants with conical geometry and morse taper connection (Alvim CM, Neodent Straumann, Curitiba, Brazil) were used in this study. Implant length and diameter were chosen based on bone availability assessed by cone-beam computed tomography.

The implants remained submerged during this period, and in the case of anterior implants, adhesively fixed bridges or removable prostheses were provided.

### Study groups

#### Control

The implant placement protocol was made following all the steps indicated by the manufacturer and according to each case.

#### LLLT group

Low-level laser (Therapy XT, DMC Group, Sao Carlos, Brazil) [Gallium Aluminum Arsenide Diode (GaAlAs)] therapy with a wavelength of 808nm wavelength, a measured power output of 50mW and a spot size of 0.4cm^2^ were applied in six points (80 s each point of application; energy density=11 J/cm^2^) prior to the preparation of the bone bed and after suturing. The application points were divided into two points in the labial region where the implant would be placed (apical and cervical): two points in the lingual region (apical and cervical) and two points in the occlusal direction. The implant placement protocol followed all the steps indicated by the manufacturer, including the sequence of the drills. The total dosage including the laser application prior to the preparation of the bone bed and after the implant placement resulted in 66 J/cm^2^. This LLLT protocol was applied only in the dental implant placement session and is based on previous studies [11, 17].

### Sample size estimation

The sample size was calculated using the inference for means tool comparing two means of the site www.stat.ubc.ca. An earlier study that assessed implant stability using the ISQ was used as the basis for this calculation [18] which reported a SD of 5.5 ISQ with expected group differences according to the bone type of 5.5 ISQ. The sample was calculated using a two-tailed comparison of two mean tests, with 90% power and 95% significance level, resulting in at least 22 implants per group. Considering possible losses, the number of included implants exceeded the sample size calculation.

### Primary outcome—implant stability

The primary outcome was the implant stability quotient (ISQ), which was assessed by means of a resonance frequency analysis device (Osstell®, IntegrationDiagnostics AB, Gothenburg, Sweden). The device was handled by a single operator and was calibrated following the manufacturer’s instructions. The smartpegs were attached to the implants, and measurements were performed in triplicate at the following intervals: baseline (T_0_—implant placement) and at the abutment selection phase (4 to 6 months, T_a_). Whenever inconsistency was observed during the ISQ assessment (e.g., lack of proper grip of the smartpegs, ISQ resulting in 0), they were excluded from the analysis. The value of insertion torque measured by the torque ratchets was also registered, in newton-centimeter (N.cm).

### Secondary outcomes—radiographic marginal bone level changes

**Fig. 1:**
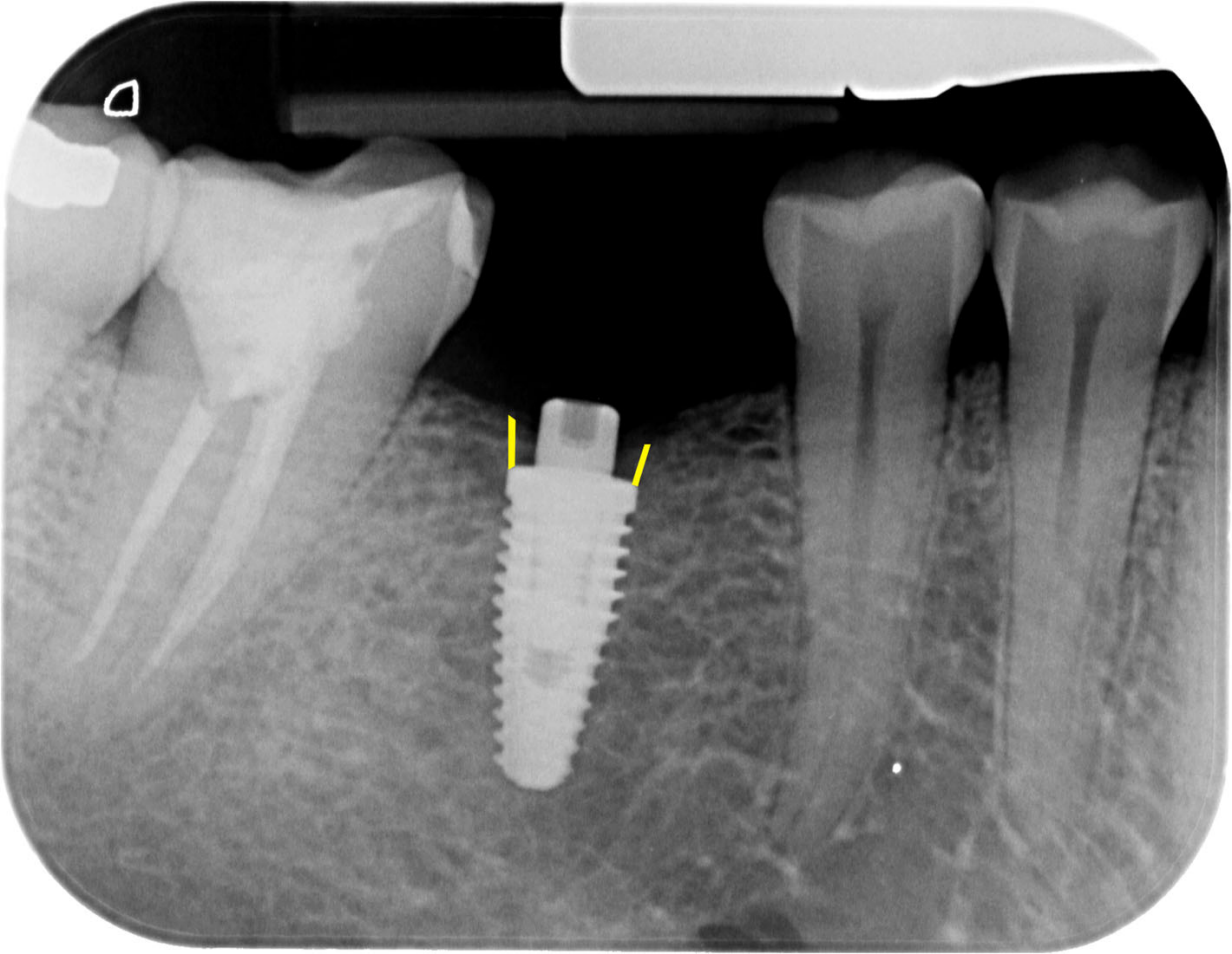
Representative image of the radiographical assessments. The distance between the implant platform and alveolar bone crest was measured both in mesial and distal regions, in millimeters, and averaged by each implant

Digital periapical radiographs were made at implant placement (T_0_) and abutment selection phase (T_a_) and were used to assess radiographical peri-implant alterations during the osseointegration period (4 to 6 months). Radiographs were performed with an intraoral X-ray positioning device by a single, previously calibrated, operator to ensure standardization. The digital files were then imported into software (ImageJ 1.47v, NIH, USA) to assess the differences in the distance between the implant platform and the alveolar bone crest in T_0_ and T_a_. The distances were measured both in mesial and distal areas (Fig. 1) and were the average value was reported according to each implant [11]. All radiographs were assessed by a single researcher who was previously calibrated and blinded to the interventions.

### Statistical analyses

Descriptive analyses with mean values and standard deviation (SD) or frequency distribution (%) were calculated for each variable. The statistical analysis was performed using Stata Software 14.2 (Stata Corporation, College Station, TX, USA). Outcome data were tested for normality by means of the Shapiro-Wilk test and found to be normally distributed. A descriptive analysis of the sample was performed, and bivariate analysis was performed to test the association between the intervention (LLLT or control) and studied outcomes using chi-square test. Possible influences of the intervention in ISQ and radiographical peri-implant alterations (T_a_–T_0_) were tested using *t* test in terms of means comparisons and its variation. Implant was considered as an analysis unit. The statistical significance was set at the alpha level of 0.05.

## Results

**Fig. 2:**
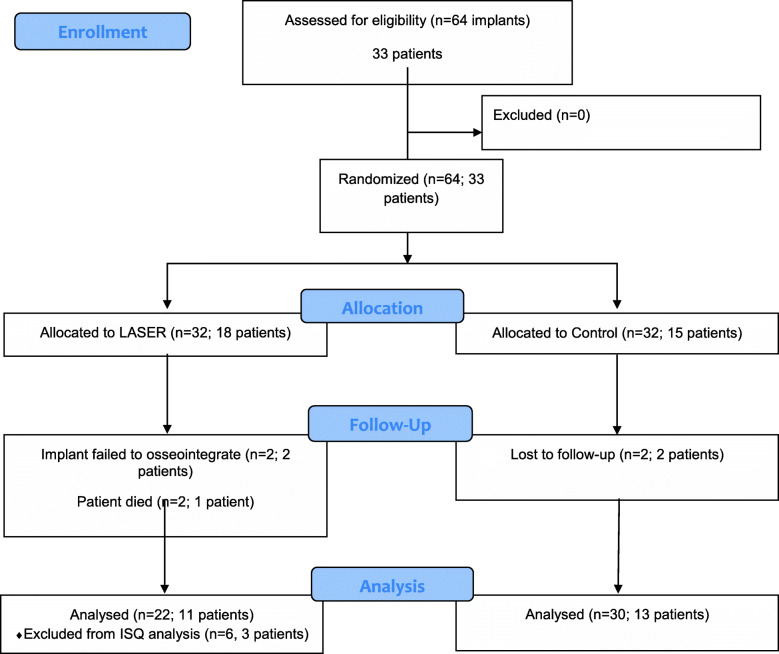
CONSORT flow diagram

A total of 64 implants were placed in 33 patients according to the randomization process. One patient of the LLLT group died from a heart attack, and then, two implants were lost to follow-up; in the control group two patients, with one implant each, were lost to follow-up. The CONSORT flow diagram with the enrollment characteristics and the number of implants and patients in each phase of the study are presented in Fig. 2.

The mean age of the sample was 49.94 years old (SD ±11.29) with a range of 27 to 70. Table 1 presents the distribution of implant and patient’s characteristics considering LLLT and control groups. A statistically significant difference was founded between groups considering hypertensive patients, where 93.7% of the control group reported the presence of controlled hypertension compared to LLLT (*p*<0.001). However, no statistically significant difference was founded between implant characteristics (region, length, and diameter), smoking habits, diabetes, age, and insertion torque between these two groups. Other general health conditions reported by the included patients were asthma (1 patient), and cardiac history (2 patients), and hepatitis C (1 patient). Two implants of the LLLT group failed to osseointegrate (3.12% of the total sample), the implants were removed, and new implants were placed in the region; however, they were excluded from the study.

**Table 1:** Distribution of implant and patient’s characteristics considering LLLT and control groups

	LLLT (***n***=32)		Control (***n***=32)		***p*** value
	***n***	%	***n***	%	
**Implant region**^a^					0.281
Anterior	12	66.7	8	33.3	
Posterior	20	43.5	24	54.5	
**Implant length**^a^					0.193
*<10mm*	18	56.3	23	71.8	
*≥10mm*	14	43.7	9	28.2	
**Implant diameter**^a^					0.777
*<4mm*	24	75.0	23	71.9	
*≥4mm*	8	25.0	9	28.1	
**Smoking**^a^					0.118
*Non-smoking*	28	87.5	32	100.0	
*Light (<10 cigarettes/day)*	1	3.2	0	0	
*Heavy (≥10 cigarettes/day)*	3	9.3	0	0	
**Diabetes**^a^					0.740
*Yes*	27	84.4	26	81.3	
*No*	5	15.6	6	18.7	
**Hypertension**^a^					0.001
*Yes*	19	59.4	30	93.7	
*No*	13	40.6	2	6.3	
	**Mean**	**SD**	**Mean**	**SD**	
**Age**^b^	49.6	11.48	50.4	11.04	0.465
**Insertion torque (N.cm)** ^**b**^	41.03	17.49	45.43	16.09	0.298

^a^Obtained using chi-square test

^b^Obtained using *t* test

No differences were observed when LLLT, and control groups were compared with ISQ difference (T_a_–T_0_) (*p*=0.598) or radiographical peri-implant alterations (*p*=0.531). However, when comparison was made between LLLT and control groups at abutment selection phase (T_a_), a statistically significant difference was found (*p*=0.030), where LLLT group presented an average distance of 1.95mm between the implant platform to the radiographic bone crest. All ISQ and implant platforms to bone crest distance values according to each intervention are presented in Tables 2 and 3, respectively.

**Table 2:** Implant stability compared between LLLT and control groups

	LLLT (***n***=22)		Control (***n***=30)		***p*** value^a^
	Mean	SD	Mean	SD	
**ISQ**					
*ISQ T*_*0*_	62.02	16.41	61.36	20.02	0.893
*ISQ T*_*a*_	62.90	11.19	65.12	8.67	0.409
*ISQ difference (T*_*a*_*-T*_*0*_*)*	0.08	18.45	2.98	20.15	0.598

^a^Obtained from *t* test.

**Table 3:** Marginal bone alterations compared between LLLT and control groups

	LLLT (***n***=23)		Control (***n***=27)		***p*** value^b^
	Mean	SD	Mean	SD	
**Marginal bone**					
Implant platform-bone crest T_0_^a^	2.05	1.32	1.64	1.28	0.270
Implant platform-bone crest T_a_^a^	1.95	1.16	1.28	0.95	0.030
Peri-implant alterations (T_0_–T_a_) ^a^	−0.89	4.33	−0.35	1.00	0.531

^a^Radiographical measures

^b^Obtained from *t* test

## Discussion

In the last decade, at least three systematic reviews covering this topic were published in the literature [13, 19, 20], and all those systematic reviews shared the positive effects of such therapy when assessed in animal models. However, all of them also emphasized the low number of primary studies in humans and highlighted the need for additional high-quality human clinical trials. In this way, our study is one of the few randomized controlled trials that assessed the influence of LLLT (GaAlAs, 808nm wavelength and 50Mw) on implant stability in implants placed in healed sites. Also, this study assessed marginal bone differences in implant placement and at healing abutment installation as a secondary outcome.

Implant stability is the main clinical factor to identify osseointegration [21], and this research field is still open to new developments. The implant stability is divided into primary (at implant placement) and secondary stability (achieved after osseointegration) [22]. Considering implants placed in the native bone, ISQ assessments suggest an increasing pattern during the healing period, which could be explained by the biological remodeling process at the implant-bone interface reflecting in the osseointegration [23]. In this perspective, our study failed to demonstrate a benefit of the LLLT compared to the control group, since the intervention groups presented lower implant stability values (ISQ) and no significant differences were observed when comparing implant stability at T_0_ and T_a_ (*p*=0.598). Thus, the null hypothesis of our study that no difference in implant stability would be observed when LLLT was compared to a control group when placing implants in healed sites was accepted. It is important to highlight that two implants of the LLLT group failed to osseointegrate (early loss), representing a 93.5% survival rate, compared to 100% of the control group. Both patients that lost these implants were heavy smokers and also had a previous history of periodontal disease, which should be considered as a limitation of our randomization process.

The peri-implant marginal bone and its alterations are another very important and reliable outcome in regard to implant success and survival [24]. It has been reported that a successful implant would not have a marginal bone loss greater than 1.5 mm in the first year [25], and in the subsequent years, it should be restricted to 0.2 mm per year [26]. As the osseointegration depends on the migration of osteogenic cells to the peri-implant surrounding area [27], it could be also hypothesized that LLLT could stimulate the early stages of bone formation. Our findings comparing the radiographical images at T_0_ and T_a_ did not present statistically significant differences (0.531). However, a statistically significant difference was observed at abutment selection phase for LLLT group (*p*=0.030), presenting an average distance of 1.95mm between the implant platform to the radiographic bone crest, similar to the recommended by the implant manufacturer (2mm), although this bone loss was in agreement with the biological process of remodeling (Table 3).

The ISQ values observed in our study presented high values of standard deviation and should be pointed out as a limitation in our study design and method since it could impair a bias-free assessment. On the other hand, the ISQ is one of the few methods that can quantify implant stability and is considered adopted as a reliable method a reproducible and reliable method that allows clinical comparisons [28, 29]. In regard to our randomization process, Table 1 showed that it was effective in distributing the implants per patients, region, and length. On the other hand, our randomization process was also not effective on distributing hypertensive patients between the groups. It is important to highlight it as a limitation; however, a systematic review on this topic failed to demonstrate the effects of hypertension on implants survival rate [30]. Also, other aspects such as bone density and quality, and the amount of residual bone are important determinants of implant stability and the non-standardization of such factors could also be considered a limitation of the present study.

Although we did not observe a positive effect of LLLT on the assessed outcomes, a recent randomized controlled trial by Dumić et al. [10] found a positive effect of LLLT to improve post-extraction bone healing, with significantly increased bone density at the follow-up compared to a control group, sonsidering that we must acknowledge that there is no consensus on LLLT protocols across the literature. Previous studies differ its methods in regard to the number of applications, wavelength, application time, and dosages. Garcia-Morales et al. [31] assessed the effect of diode-laser (830nm diode-laser, total of 5 J/cm^2^) applied in 8 sessions (1 postoperative and 7 additional irradiations) and other studies Torkzaban et al. [12] used longer wavelengths of diode-laser (940 nm, total of 8 J/cm^2^) applied in seven sessions, while Matys et al. [14] investigated the use of lower wavelengths of diode-laser (635 nm) with a single application prior to implant placement and five additional sessions (immediately after surgery, 2, 4, 7, and 14 days). Our present study adopted a different approach for LLLT application [11], with a first application in the bone site prior to the preparation of the bone bed and a second application after suturing, with a total dosage of 66 J/cm^2^. One could suppose that a single LLLT session would not have a significant biostimulation effect compared to protocols with more LLLT sessions. However, none of these studies, regardless of the number of sessions, showed a positive effect of LLLT on the implant stability. On the other hand, the positive effect of LLLT on implant stability and in the bone cell proliferation was observed in in vitro studies using both single and multiple LLLT sessions, and different wavelengths [19, 32]. A possible explanation for these contradictory findings is that human bone metabolism is not sufficiently affected by the amount of energy that could improve bone metabolism in animal models. Therefore, it would be recommended to focus research efforts on customizing the LLLT protocols for human bone metabolism rather than just rejecting the functionality of such therapy when placing dental implants.

## Conclusion

Within the limitations of our study, our findings suggest that LLLT applied in healed bone sites before the preparation of the bone bed and in the surgical wound after suturing have no positive influence in the implant stability compared to a control group.

## Data Availability

Not applicable
